# A complete workflow from embalmed specimens to life‐like 3D virtual models for veterinary anatomy teaching

**DOI:** 10.1111/joa.14192

**Published:** 2024-12-20

**Authors:** Zeeshan Durrani, Fay Penrose, James Anderson, Emanuele Ricci, Stephanie Carr, Lorenzo Ressel

**Affiliations:** ^1^ Department of Veterinary Anatomy, Physiology and Pathology, Institute of Infection, Veterinary and Ecological Sciences University of Liverpool Liverpool UK

**Keywords:** 3D models, anatomical specimens, anatomy education, anatomy resources, cadaver dissection, cadaver embalming, photogrammetry

## Abstract

Understanding normal structural and functional anatomy is critical for health professionals across various fields such as medicine, veterinary, and dental courses. The landscape of anatomical education has evolved tremendously due to several challenges and advancements in blended learning approaches, which have led to the adoption of the use of high‐fidelity 3D digital models in anatomical education. Cost‐effective methods such as photogrammetry, which creates digital 3D models from aligning 2D photographs, provide a viable alternative to expensive imaging techniques (i.e. computed tomography and magnetic resonance imaging) whilst maintaining photorealism and serving multiple purposes, including surgical planning and research. This study outlines a comprehensive workflow for producing realistic 3D digital models from embalmed veterinary specimens. The process begins with the preservation of specimens using the modified‐WhitWell (WhitWell‐Liverpool) embalming protocol, which ensures optimal tissue rigidity and improved colour enhancement, facilitating easier manipulation and better photogrammetry outcomes. Once embalmed, specimens are photographed to create digital 3D models using photogrammetry. Briefly, all images are processed to generate a sparse point cloud, which is then rendered into a 3D mesh. The mesh undergoes decimation and smoothing to reduce computational load, and a texture is applied to create a lifelike model. Additional colour enhancements and adjustments are made using digital tools to restore the natural appearance of the specimens. The 3D models are stored on a cloud repository and integrated into the University of Liverpool's Virtual Learning Environment, providing continuous, remote access to high‐quality anatomical resources. The switch to embalmed specimens during the COVID‐19 pandemic allowed for longer‐term use and detailed dissections, enhancing the quality of digital models. Fresh specimens, though naturally coloured, are less stable for photogrammetry, making embalmed specimens preferable for accurate 3D modelling. Our method ensures embalmed specimens are rigid enough for precise modelling while allowing texture adjustments to enhance digital representation. This approach has improved logistical efficiency, educational delivery, and specimen quality. Innovative embalming techniques and advanced photogrammetry have the power to revolutionise anatomical education with the creation of a vast digital library accessible online to students at any time. This approach paves the way for integrating digital 3D models into immersive environments and assessing their impact on learning outcomes.

## Graphical abstract

This study introduces a workflow using the Liverpool‐Whitwell embalming protocol and photogrammetry to create realistic 3D digital models from veterinary specimens. These models, enhanced with digital tools and stored in a cloud repository, are integrated into the University of Liverpool's Virtual Learning Environment, offering remote access to high‐quality anatomical resources. This method enhances educational delivery, specimen quality, and logistical efficiency, revolutionising anatomical education and improving learning outcomes.

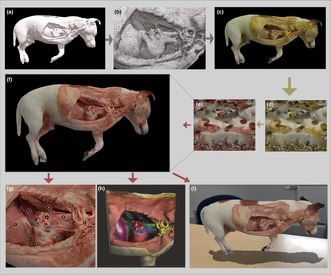

## 1 INTRODUCTION

Learning structural and functional anatomy is a core aspect of the curriculum for numerous health professionals, including medical, veterinary and dental courses (Hackmann et al., 2019). Application of anatomical knowledge, including organ recognition and topography, provides the foundation for clinical examination and surgical training and allows students to understand the pathogenesis of diseases (Cheung et al., 2021; Marks, 2001; Turney, 2007). Prior to the COVID‐19 pandemic, Liverpool Veterinary School relied on fresh‐frozen cadaver‐based dissections for all anatomy teaching. However, due to a shortage of cadavers and pandemic‐related disruptions, the teaching strategy was abruptly altered to accommodate the demand for cadavers and ensure socially distanced, face‐to‐face instruction. Consequently, robust preservation methods were introduced to maintain cadaver longevity and appropriateness for gross anatomy teaching across multiple years and programmes. Additionally, numerous pressures have recently led to a reduction in the time allocated to face‐to‐face cadaver‐based anatomy teaching within contemporary curricula (Theoret et al., 2011). These include reductions in funding and staff, increased class sizes, increasingly restrictive legislation, and ethical challenges (Santana et al., 2022; Theoret et al., 2011). These considerations, together with pedagogical development in the application of blended learning, integrating authentic online learning with traditional in‐person sessions to improve student learning experiences, have subsequently led to the development, and greater involvement, of three‐dimensional (3D), high‐fidelity digital models for anatomy teaching (Davis & Fill, 2007). In addition to student education, digital models hold numerous other potential applications, including macroscopic examination of biopsies and surgical resections, surgical planning and research (Hong et al., 2021; Turchini et al., 2018).

Outside of the dissection room, understanding 3D anatomical structures through a reliance on 2D, often passive, teaching materials can be challenging, while the use of 3D digital models has been shown to improve significantly subject comprehension among students (Gilbert, 2004; Wesencraft & Clancy, 2019). Students learn better when they are actively engaged, with interactive student‐centred learning leading to improved outcomes (Russell et al., 2016). This is central in the development of contemporary curricula, highlighted within University College London's Arena Blended Connected Learning Design (ABC LD) model, based on Diana Laurillard's educational framework (Laurillard, 2002; Young & Perović, 2020). Students utilising active learning strategies, such as 3D digital resources, as well as augmented and virtual realities, demonstrate stronger improvement in their anatomical knowledge than individuals focusing on passive learning activities, such as 2D and text‐based resources (Langfield et al., 2018; Little et al., 2018; Maresky et al., 2019; Zilverschoon et al., 2019). Recent publications have also indicated that staff and students preferred the ‘high realism’ of digital 3D models to less realistic models for preparation and knowledge consolidation following anatomy practical classes. Colour‐coding (using bright colours to highlight anatomical structures) of digital 3D models was associated with improved anatomy performance whilst not significantly increasing the cognitive load of learners (Erolin, 2023; Koh et al., 2023). Our Department recently received positive feedback from students following the inclusion of digital 3D models during veterinary pathology teaching, with over 85% of surveyed students finding the models engaging and informative, enabling them to understand better the lesions presented (Ricci et al., 2024).

Among different approaches, digitalised 3D models are produced through a process called photogrammetry (Egels & Kasser, 2002). This technique models a 3D space by collecting geometric information via a series of overlapping two‐dimensional (2D) still image photographs taken from numerous angles. This method is relatively inexpensive to undertake, requiring only a camera, appropriate lighting and photogrammetry software (Rajaram et al., 2023). Financially, it is therefore a more viable option than considerably more expensive methods, such as computed tomography (CT) imaging and magnetic resonance imaging (MRI), or professional lidar or laser 3D scanners, whilst also maintaining the photorealism of the original specimen (de Lima et al., 2019).

Ideally, to accurately capture the realism of an anatomical specimen containing soft tissue, digitalised models would predominantly focus on fresh material. However, this methodology can present numerous challenges (Titmus et al., 2023). Fresh, wet specimens deteriorate over relatively short periods of time, are soft and thus flexible, leading to challenges in obtaining full 3D reconstructions when suspended or moved while capturing photographs from multiple angles (requiring stabilisation). In addition, the high moisture content of fresh specimens results in shiny surfaces that hinder the ability of photogrammetry software to identify sufficient common alignment points for the generation of a single polygonal mesh required during processing (Nicolae et al., 2014; Titmus et al., 2023). Specimens can however be preserved, often using formalin as a fixative agent for whole organs, selected soft tissue samples, or through embalming an entire cadaver (Lombardero et al., 2017). This methodology results in an increased rigidity of the sample, allowing easier sample manipulation that, together with increased sample opacity, ensures consistency in the acquired photographs with reduced surface reflection (Ling et al., 2016). In addition, these properties offer greater time to conduct lengthy and detailed dissections of specimens, ultimately producing more complex and informative digitalised models for learning purposes. However, embalmed biological tissues lose their natural colouring, deteriorating over time, giving them artefactual colours, subsequently reducing the realism of the original specimen (Lombardero et al., 2017). When generating a 3D digital model, these colours can be recovered through digital enhancement, reinstating the realism of the original fresh specimen, or even augmenting natural colours, adding layers for better characterisation of areas. Finally, models can be embedded into virtual environments such as augmented reality (AR) (Gurses et al., 2024), virtual reality (VR) (Krause et al., 2023) or mixed reality (MR) (Kumar et al., 2021), altogether referred to with the umbrella term “Extended Reality” (XR), to increase the immersive component of the learning experience.

The aim of this article was to illustrate a workflow for the production of more robust veterinary anatomy teaching resources that contain more realistic colouring, utilising both our novel embalming and 3D modelling methods. We have therefore described our current workflow for producing high‐quality, realistic 3D digital models from preserved veterinary anatomy specimens using our WhitWell‐Liverpool embalming protocol, acquisition of photographs, creation of 3D digital models and texture enhancement and embedding into web‐based viewers or immersive environments.

## 2 MATERIALS AND METHODS

### 2.1 Embalming

Veterinary anatomical teaching utilises a wide range of species, including canine, feline, ovine, equine, and bovine specimens. However, for the sake of clarity and focus on this paper, we will primarily discuss canine and feline species, as they are the core models used for teaching anatomy. Since 2020, we have embalmed over 65 canine and 15 feline cadavers, which are actively used as prosected specimens in both Year 1 and Year 2 anatomy teaching. Cadavers were usually frozen within 12 h after death and subsequently slowly thawed for a minimum of 3 days in cold room facilities (4°C) prior to the embalming procedure. Occasionally, fresh cadavers (within 48 h of death), which were stored in a cold room, were also used for embalming.

Since 2019, we have collaborated with Ben Whitworth (MazWell Group Ltd., Whitchurch, Hampshire, UK), a specialist in developing embalming protocols for veterinary and medical schools to enhance anatomical education. Over the past 4 years, we have adopted and refined a novel embalming protocol known as the WhitWell‐Liverpool method, which incorporates a tailored chemical mixture, primarily using Dodge embalming products (Dodge Co., Billerica, MA, USA). This method employs a combination of specialised equipment, tools, and a custom mix of chemicals to meet the specific needs of our anatomy teaching facilities. The primary goal of this protocol was to enhance cadaver preservation by improving tissue longevity, texture, colour, and odour, while also considering time efficiency, cost‐effectiveness, sustainability, and safety. For the preservation of whole cadavers, we utilised a combination of embalming fixatives designed to maintain the natural consistency of fresh tissues and enhance colour retention compared to conventional embalming methods. A comprehensive list of the fixatives used is provided in Table S1.

A pump flow system was utilised in this protocol to allow for a steady tissue perfusion through pulse administration with controlled pressure and flow rate, which is superior to older pumps or gravity feed methods. This system allows for quicker and more uniform perfusion, taking approximately 60–75 min per cadaver. To achieve de‐coagulation and stabilise blood pH, a pre‐embalming mix was injected into the vascular system, consisting of Proflow and Rectifiant 1:1 (Dodge Co., Billerica, MA, USA). This mix facilitates the dissolution of blood clots, restoring cellular permeability and fluid balance. The embalming process described involves three key steps: first, a pre‐embalming mix is perfused for 10–15 min to condition the tissues and improve fluid distribution. This is followed by a 20‐min resting phase, allowing the solution to diffuse throughout the body. Finally, the primary embalming solution is injected to preserve the tissues, prevent decomposition, and restore a natural appearance.

The embalming mix contains Proflow (1 L), Rectifiant (2 L), Restorative (1 L), Dispray (1.5 L), Halt GX (0.5 L), and Introfiant (3 L) (Dodge, Billerica, MA, USA). This mixture results in a total of 9 L for a cadaver weighing approximately 25–40 kg (see Table S1). For smaller cadavers (15–25 kg), the volume of introfiant is reduced by 1 L. A flow rate of 300–400 mL/min is maintained with a pulse pressure of 120–140 psi.

In comparison to the previously published Newcastle‐WhitWell protocol (Thompson et al., 2022), we have incorporated Dodge's latest pre‐ and co‐injection chemical, Proflow, which functions as a high‐performance wetting agent and humectant, offering superior flow and colour distribution. The advanced formulation of Proflow, compared to Metaflow, enhances vessel lubrication, operates effectively across a broader pH range and water hardness conditions, and facilitates deeper penetration of the arterial solution into tissues. Additionally, we improved preservation by introducing Halt GX, a new co‐injection chemical designed to inhibit gas production. Halt GX is particularly effective in cadavers showing early signs of decomposition and abdominal distension. Specimens embalmed with Halt GX exhibited better resistance to the development of mould and maintained a slightly firmer texture.

**FIGURE 1:**
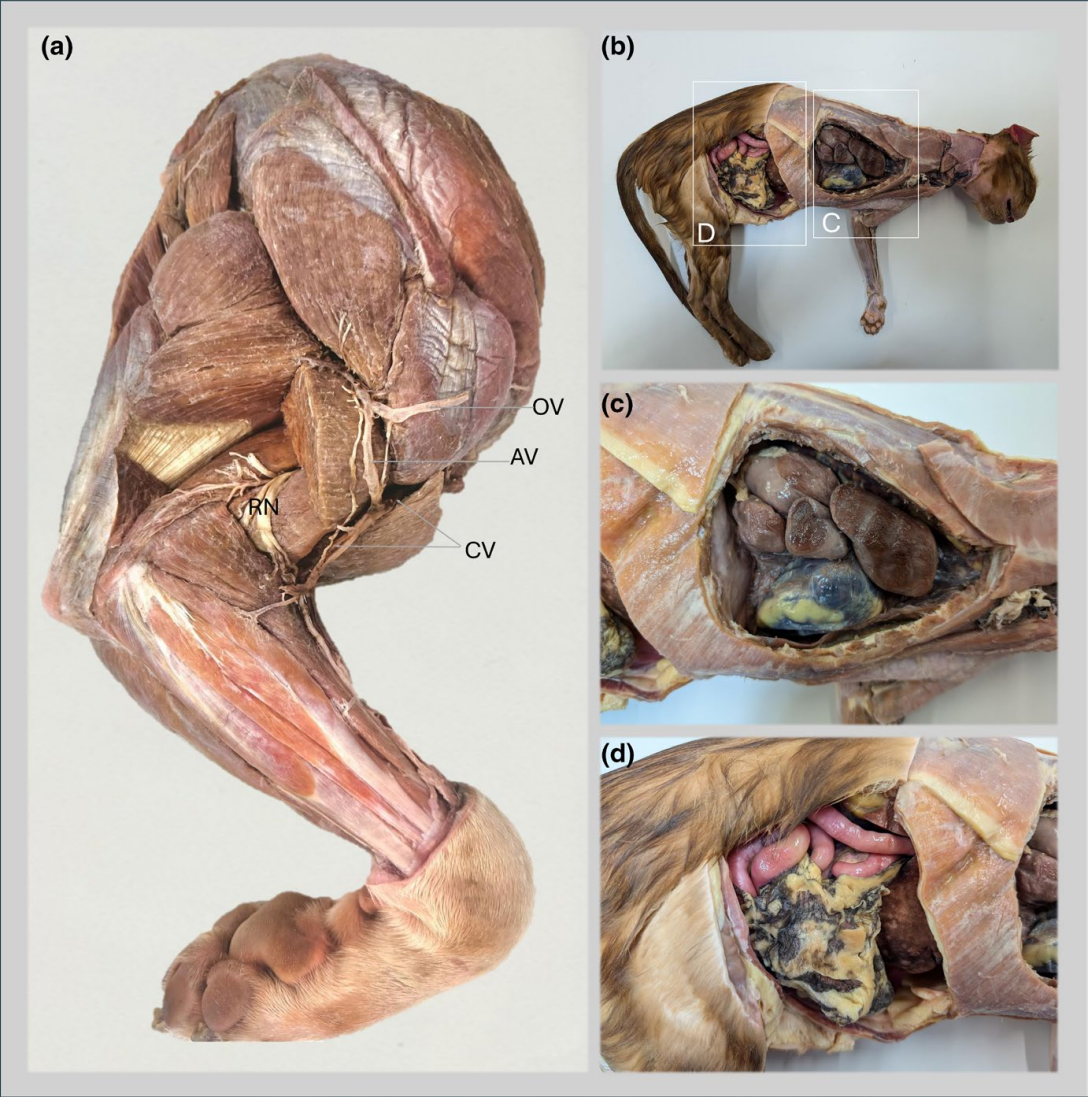
Representative images of dissected specimens embalmed using the WhitWell‐Liverpool method, illustrating the preserved condition of the organs and their appearance. (a) Lateral view of the right thoracic limb of a dog showing muscles of the brachium and antebrachium, nerves, and blood vessels. The lateral head of the triceps is removed to reveal the radial nerve and deeper muscles. The superficial vein system, including the cephalic vein (CV), omobrachial vein (OV), and axillobrachial vein (AV), is also shown. (b) Embalmed cadaver of a cat showing the right lateral view of the dissected thoracic and abdominal cavities with various organs in situ. (c) Thoracic wall partially removed to show the lobes of the right lung and the heart within the fibrous pericardium. (d) Lateral abdominal muscles removed to show various organs in situ. Note the preservation of dorsally placed large intestine (pink) and adipose tissue in the greater omentum, covering the ventrally placed small intestine.

Monitoring the success of embalming involved ensuring full uptake of fluid without loss through the nares or other orifices, while detecting abdominal swelling and rigidity in the limbs and tail together with light pink discolouration of the ventral abdominal skin and auricular pinnae, all of which together indicate adequate perfusion. Limbs and extremities were constantly manipulated throughout the process to facilitate embalming fluid perfusion. The embalming process continued until all tissues were adequately coloured and filled. If perfusion was inadequate, additional embalming fluid was introduced via a local cannulated artery. Upon completion of the procedure, the abdomen should appear distended, while the limbs and tail should appear rigid. The embalmed specimens were wrapped in transparent polythene bags and stored for a minimum of 2 months to allow for complete curing of the tissues. An experienced prosector carefully performed dissection of all embalmed cadavers on a downdraft table to ensure safe manipulation and inspection. Examples of embalmed specimens are presented in Figure 1. Selected embalmed specimens then underwent photogrammetry. The Research Ethical Committee of the University of Liverpool has granted approval for the use of anatomical specimens and cadavers for teaching and research purposes (University of Liverpool, Veterinary Research Ethics Committee RETH000553/VREC480).

### 2.2 Photogrammetry and image processing

**FIGURE 2:**
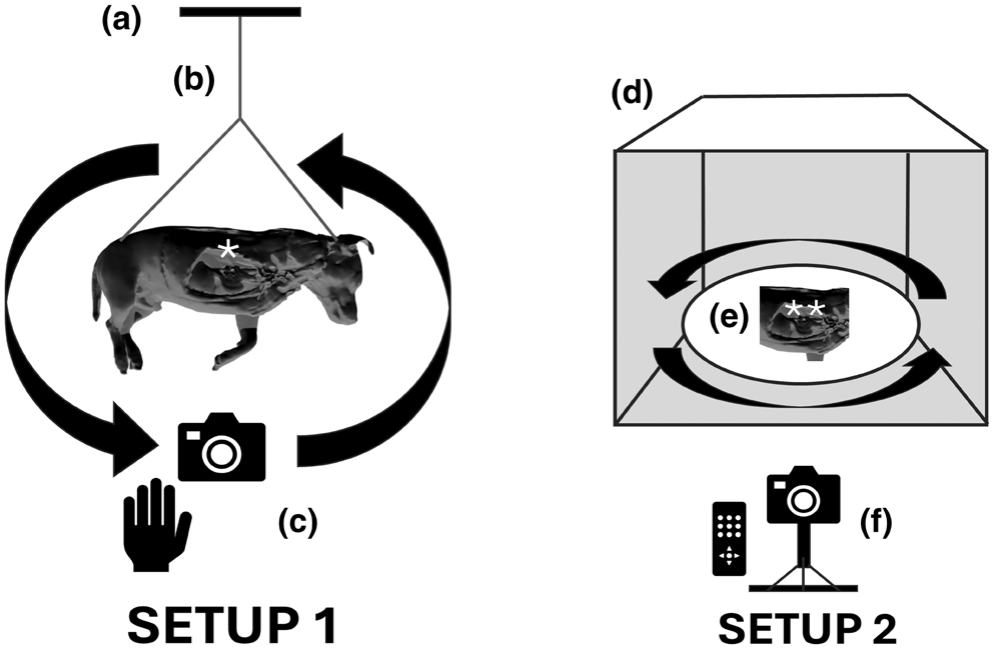
Schematic drawings of the two photogrammetry setups used. Setup 1 (left) is intended for suspended specimens, such as an entire cadaver or large body parts. The specimen (*) is securely suspended from the ceiling at point (a) using a strong, clear nylon fishing line (b), and once the specimen is completely static, the image capture process begins. A hand‐held camera (c) is moved around the specimen in circular patterns at different heights. Setup 2 (right) is designed for relatively small and rigid specimens. The specimen (**) is placed on a rotating platform (e) within a controlled environment, such as a photo booth (d). The controlled lighting and backdrop create optimal light conditions. The rotating platform gradually turns the specimen, enabling sequential capture from various angles. A fixed, remotely controlled camera (f) takes images as the platform rotates. To ensure that all surfaces are captured, the specimen will need repositioning throughout the process.

**FIGURE 3:**
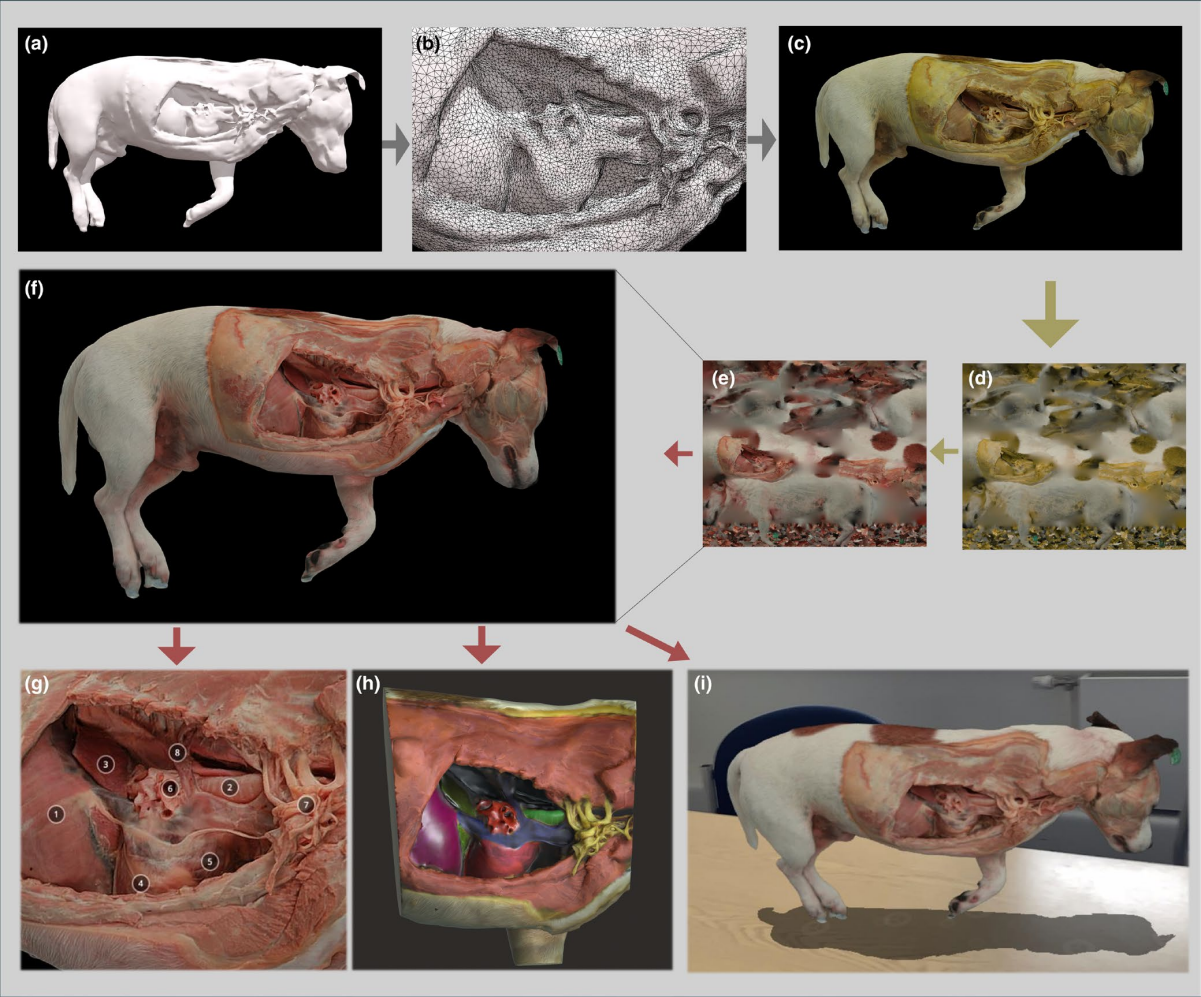
Workflow of embalmed cadaver digitalisation illustrated on a full canine body specimen with the right lateral wall of the thoracic cavity prosected. (a) Mesh obtained from the point cloud after alignment of photographs; (b) decimation of vertices, showing the wireframe. (c) Mesh with texture applied from the original embalmed specimen; (d) unwrapping of the texture. (e) Processing of the native texture with alteration of colours and restoration of natural colours; (f) final 3D model with altered colour texture applied; (g) application of pinpoint annotations to highlight specific structures; (h) painting of different structures using vertex colours; (i) embedding and visualisation of the model in an augmented reality application.

Embalmed specimens, either full cadaver or body parts, were used for our photogrammetry workflow. Large and rigid samples were suspended using transparent nylon fishing wire (0.45 mm diameter) from the ceiling (Setup‐1) and smaller specimens were positioned inside photo booths (Setup‐2) as explained in Figure 2. In Setup‐1, multiple photographs (200–600) were taken by the operator following circular paths, taking a picture approximately every 5°, around the object and repeating the same process at different heights, as previously described by us (Ricci et al., 2024), and for other photogrammetry applications (de Lima et al., 2019; de Oliveira et al., 2023; Dixit et al., 2019; Evin et al., 2016; Struck et al., 2019; Talevi et al., 2023). In Setup‐2, a fixed camera was placed at a specific position relative to the light box containing the sample. The object was rotated on a turntable with consecutive photographs taken at incremental intervals (every 5°) by a fixed camera. This process continued until the object had rotated 360°, resulting in a series of overlapping 2D photographs taken from different angles. The object orientation was then changed on the turntable and the process repeated. For this study, a Nikon Z50 camera (Nikon, Tokyo, Japan) equipped with a 50 mm Macro lens (50 2.8 MC, Nikkor, Nikon, Tokyo Japan) was used. Using the pictures obtained (13.8 MP; 3712 × 3712, .jpg format), specific features of the object were translated into a sparse point cloud via photogrammetry software (Reality Capture, Capturing Reality, Epic Games, Cary, North Carolina) and rendered into a solid 3D texture‐less mesh (Figure 3a). The obtained mesh, which natively is approximately several million polygons, was then subjected to further processing, in particular, polygon decimation (with a target of 100,000–150,000 polygons) and smoothing of the 3D mesh (Figure 3b), which reduces the computational effort required within the subsequent phases.

A texture, resulting from the merging of the colour information extracted from the original set of photographs, was applied to the 3D surface to create a textured model (Figure 3c). The texture relative to the model (available as a .Jpeg file—Figure 3d) was uploaded into Adobe Photoshop (Adobe, San Jose, California, USA). The image was processed using the “selective colour adjustment” function. Using the dropdown menu labelled “colours,” which contains options such as Reds, Yellows, Greens, Cyans, Blues, Magentas, Whites, Neutrals, and Blacks, the image was modified using normal tissue colours from photographs of fresh specimens as a comparison until they reached the most likely resemblance to real‐life colours. Using our current embalming technique, the following standard settings have provided the best result for most tissues processed: Reds (Absolute: Cyan −13; Magenta +4; Yellow −49; Black +5); Yellows (Absolute: Cyan −88; Magenta +100; Yellow +9; Black 0); Greens, Cyans, Blues, Magentas, Whites, Neutrals, Blacks: unchanged (0). However, additional ad hoc small adjustments to match the colours of the original fresh tissues were in some cases needed due to slight variation in camera exposure and lighting condition during photographic acquisition. More drastic adjustments are likely needed if different embalming protocols are used. In some circumstances, the “select” function in Adobe Photoshop can be used to alter the colours of different portions of the texture selectively. The processed texture image was then obtained (Figure 3e) and re‐applied over the colourless 3D mesh to obtain a life‐like specimen model (Figure 3f; Video S1). Once completed, textured 3D meshes can be saved in common 3D file formats such as “.obj” or “.glb”. The obtained model can be further processed using trimming functions in slicer software (Meshmixer, Autodesk, San Rafael, California, USA) to focus on specific portions and visualised in cloud 3D viewers such as Sketchfab (Sketchfab, New York, USA), which allow further editing and in particular annotation of selected structures (Figure 3g). The final model, exported to a 3D painting and sculpting software (Nomad Sculpt, Hexanomad), can be further edited by applying artificial colours to vertices (vertex colours) to highlight specific structures as an alternative or addition to pin‐point annotations (Figure 3h). In this instance, it is recommended to not decimate the model too aggressively, keeping the number of polygons sufficiently high, ensuring good visualisation of the artificially coloured model. Once finalised, the model can be embedded in the 3D viewer of the selected platform (Sketchfab, New York, USA) to visualise it on the screen or via an AR‐enhanced viewer (Sketchfab, New York, USA), where the model can be seen together with its natural environment through the screen of a tablet or mobile phone (Figure 3i).

### 2.3 Maintaining the digital library and integration of the 3D anatomical models into the virtual learning environment (VLE)

The entire collection of digital 3D models is maintained in SketchFab 3D viewer, which offers a wide range of functionalities for model enhancement and interactive exploration. Once uploaded to SketchFab, the 3D models are made available to students via HTML code embedding, through the university's virtual learning environment (VLE), which uses the CANVAS platform (Instructure, Salt Lake City, USA). This allows restriction of view to members of the school in order to avoid dissemination of potentially sensitive images to the public. Dedicated folders were created within CANVAS for all anatomical 3D models, each associated with specific teaching sessions focused on separate body systems. Anatomical specimen commentaries and practical worksheets were also provided to aid students in accurately interpreting and identifying anatomical structures. Integrating 3D models into the VLE ensured that students have continuous and safe access to high‐quality anatomical resources, facilitating an enhanced learning experience regardless of physical classroom constraints.

### 2.4 Course and student information

At the University of Liverpool School of Veterinary Science, anatomy is primarily taught during the pre‐clinical years to students enrolled in the Bachelor of Veterinary Science (BVSc) program. The cohort typically consists of approximately 175–210 students. During the first 2 years of the program, students study the normal structure and function (NSF) of all major body systems in both common and exotic species. The student population is predominantly female, comprising over 80% of the cohort, with male students making up 15%–19%. Additionally, mature students represent 11%–15% of the cohort, reflecting a diverse range of backgrounds and experiences within the student body.

## 3 RESULTS AND DISCUSSION

In the current manuscript, we present the practical details of a newly designed workflow for the creation of digital 3D models of canine and feline embalmed cadavers for use in veterinary medicine undergraduate teaching.

Several methods are used to preserve cadavers for anatomical study, including traditional fresh‐frozen cadavers, Thiel embalming, phenoxyethanol or formalin‐based embalming, ethanol‐glycerine embalming, saturated salt fixation, and plastination (Coleman & Kogan, 1998; Fruhstorfer et al., 2011; Hayashi et al., 2016; Lombardero et al., 2017; Lone et al., 2017; Tamayo‐Arango & Garzón‐Alzate, 2018), which are well described by Brenner (2014) and McLachlan and Patten (2006). Each method has unique advantages and disadvantages, affecting factors such as tissue colour, odour, feel, flexibility, storage requirements, toxicity, safety, and preservation longevity (Brenner, 2014). These factors significantly influence the quality and effectiveness of anatomical education. In the past, we have used fresh‐frozen cadavers, and based on our experience, they clearly retain the natural colour and feel of the tissue. However, these specimens require specialised storage, have limited preservation duration, and carry potential health risks from putrefaction. Additionally, using fresh anatomical specimens for digitisation and 3D modelling can be problematic, as certain highly flexible organs may not maintain a static shape, leading to image distortion.

The introduction of the WhitWell‐Liverpool protocol has brought significant benefits to our veterinary anatomy course, enhancing logistics, course delivery, educational experience, and maintaining the quality of anatomical specimens compared to previous methods. This protocol enables us to preserve cadavers with superior qualities suitable for both prosection‐based teaching and the production of 3D digital models. This has enhanced our pedagogical strategies for teaching veterinary anatomy within our department, engaging in a multimodal and blended learning approach and integrating conventional and face‐to‐face teaching methods with digital technologies (Tayebinik & Puteh, 2012; Varga‐Atkins, 2024). Unlike previous formalin‐phenol‐based practices, the WhitWell‐Liverpool method offers improved tissue colourisation due to the presence of dye, enhanced tissue feel, and good durability of delicate organs such as the lungs, liver, and gastrointestinal tract (Figure 1). Overall, excellent results are achieved when blood and clots are well‐drained during the pre‐fixation stage. This protocol better preserves organs with high enzymatic contents, such as the liver and kidneys, when compared to methods using saturated salt or Thiel's solution (Lombardero et al., 2017). Muscles retain improved colouration and rigidity, while nerves and vasculature remain movable in situ. Dissecting these specimens allows the separation of fascial planes and long‐term preservation, preventing fungal or bacterial growth and spread within and between cadavers in the teaching and dissection spaces. Similar results are reported by Thompson et al. (2022), who used the Newcastle‐WhitWell protocol as a parallel pilot approach yet slightly different version of the WhitWell‐Liverpool in human cadavers at Newcastle Medical School.

Whilst fixed specimens are generally more challenging to dissect than fresh specimens, the longevity of the material allows for more detailed dissections to be completed and the material to be reused on multiple occasions. The WhitWell‐Liverpool embalming technique produced specimens that were somewhat less rigid and more pliable than traditional high‐formalin‐level embalming solutions produce. With traditional high‐formalin solutions, tissue planes were easily separated, although muscle fibres were somewhat friable and prone to tearing if not handled delicately. Colour preservation was generally good, although it did tend to fade after prolonged exposure to air and repeated handling. While the tissue using the WhitWell‐Liverpool protocol is less rigid than traditional formalin‐based fluids, it is rigid enough to preserve position if moved or rotated, rendering the specimens suitable for subsequent use in digitisation via photogrammetry.

At Liverpool School of Veterinary Science, we have recently established a photogrammetry workflow that is utilised for digitising both veterinary pathological specimens (Ricci et al., 2024) and gross anatomical specimens. In veterinary pathology, most of our specimens are fresh and photographed during post‐mortem examination. This approach preserves the natural colour of the specimens and eliminates the need for post‐processing to restore colours. However, utilising fresh specimens for photogrammetry presents several challenges. One primary limitation is the lack of appropriate rigidity in fresh specimens, which is necessary to maintain stability during photography. For comprehensive full 3D capture, the specimen must remain stable, but fresh tissues are inherently flexible and prone to deformation. As a result, most of our fresh specimens are digitised lying flat, minimising movement caused by gravity and air movements, which can alter the position of organs and other anatomical structures during capture. While the flat‐lying approach helps to prevent distortions, it does not allow all aspects of the specimen to be captured, including the natural position of the body part in space. The inability to keep specimens in a relatively fixed position without deformation leads to significant challenges in mesh generation. When parts of a specimen move during capture, it can result in the creation of multiple different meshes instead of a single coherent one. This loss of accuracy and coherence in the creation of 3D digital models poses significant challenges and complications.

In the context of pathology, where specimens are typically presented on the surface of the necropsy table, digitising specimens on a flat surface does not pose a significant issue. However, this approach presents substantial limitations for the digitisation of gross anatomical specimens. In gross anatomy, understanding the full 3D appearance of the organ is crucial for learning outcomes, and the inability to capture the specimen from all angles, without deformation, may compromise the educational value of the digital model. Thus, while fresh specimens offer the advantage of preserving natural colour and avoiding post‐processing, their lack of rigidity and subsequent challenges in maintaining stability during photography significantly hinder the creation of accurate and comprehensive full 3D models, particularly for gross anatomical education. Additionally, the ability of further processing the texture with artificial colours offers an unlimited resource for creating educational models targeted for the comprehension of a specific area, organ or system, starting from a single original sample. This strengthens the value of our 3D digital models to our students, given that ‘high realism’ digital 3D models are preferred to less realistic models to prepare for and consolidate knowledge after gross dissection practical classes and that colour‐coding of 3D models has been shown to improve anatomy performance whilst not significantly increasing the cognitive load (Erolin, 2023; Koh et al., 2023).

Our current photogrammetry protocol is relatively fast, averaging around 1.5 h in total per specimen (30 min photocapturing, 1 h image processing and editing), with an additional 30 min for further post‐processing when needed. The time needed for processing is however strictly dependent on CPU and GPU power used and can stretch to several hours if suboptimal hardware is employed. Despite the other techniques to create 3D models from objects, photogrammetry seems to have advantages in this particular context. As an example, one of the main advantages of photogrammetry over laser scanning, particularly when dealing with small, detail‐rich objects, is cost‐effectiveness. Photogrammetry generally requires only a digital camera and appropriate lighting and photogrammetry software, making it significantly cheaper than laser scanners, which can be quite expensive due to their complex hardware requirements (Remondino et al., 2012). In addition, there are several photogrammetry software packages that are released as freeware at present.

**FIGURE 4:**
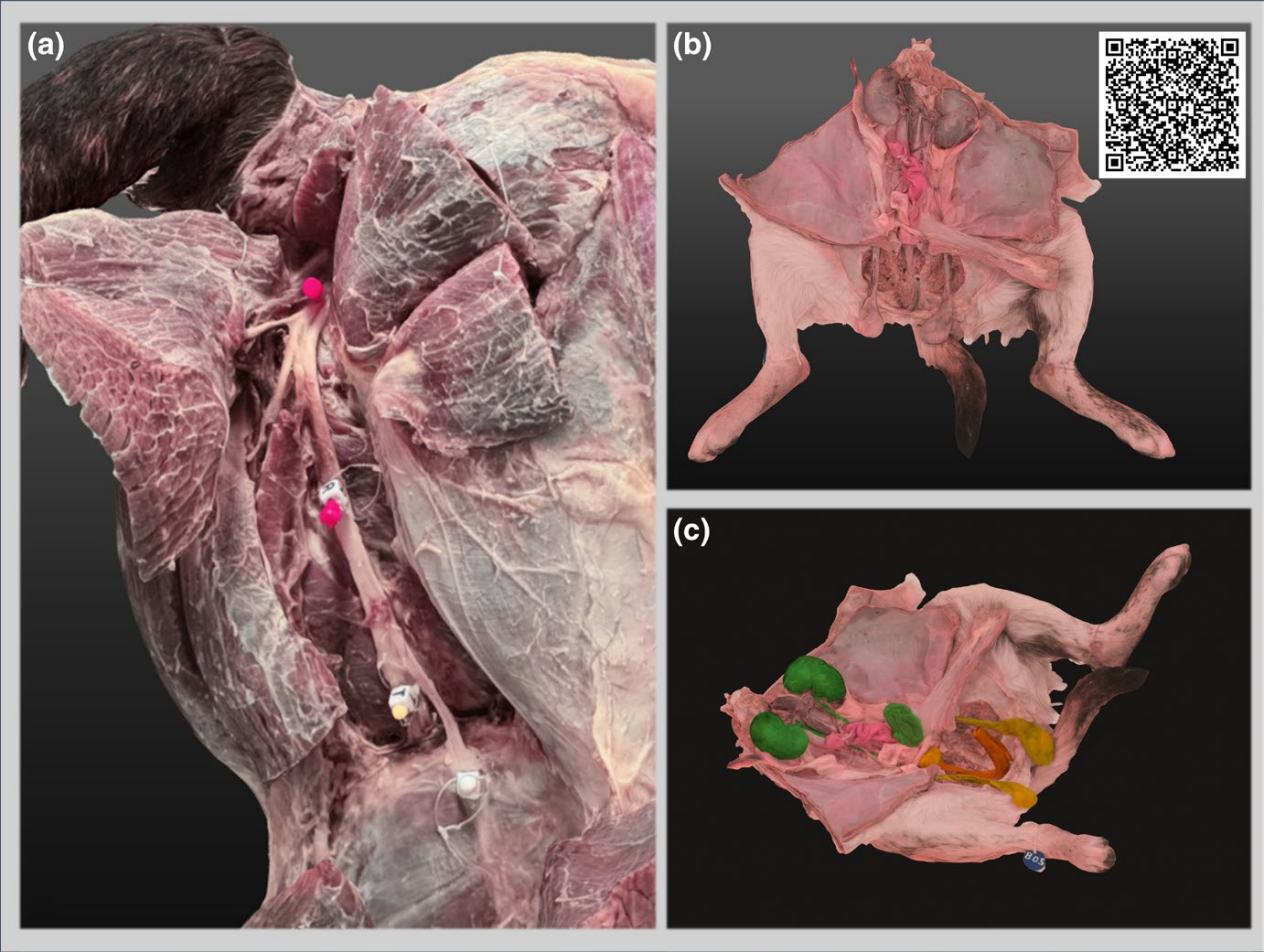
Examples of anatomical specimens transformed into 3D digital models with software‐assisted transformation and colour enhancement for improved anatomical understanding. (a) Pelvic limb specimen of a dog showing muscles and nerves, with individual nerves identified by coloured pins (note the colour enhancement for realistic appearance). (b) Hindquarter of cadaver showing exposed male urogenital organs. Inset: QR code to access the model. (c) Colouration of individual organs and structures within the urogenital system: Kidneys, ureter and urinary bladder (green), testes and spermatic cord (yellow), and penis (orange).

We have developed a strategy for intuitive access, visualisation, rotation, and annotation of 3D models on any computer device, enhancing student interactivity and experience. Figure 4 represents examples of 3D digitised models, including an annotated, non‐transformed embalmed specimen, focusing on the pelvic limb (Panel A), the hindquarter of an embalmed specimen with life‐like colour transformation (Panel B), and its counterpart painted version (Panel C) where specific anatomical structures are highlighted. Using Sketchfab as a cloud‐based repository for 3D models offers significant advantages over local storage and hardware solutions (e.g., GPU requirements). Sketchfab allows users to store, view, and share 3D models online from anywhere with an internet connection, improving accessibility for learning. It also facilitates seamless collaboration, as multiple users can access and visualise models simultaneously, regardless of their location. Additionally, Sketchfab provides powerful visualisation and presentation tools, including native support for AR, which are typically unavailable with local hardware setups. The platform employs robust security measures to protect user data from unauthorised access and cyber threats. Its integration capabilities with various 3D software and the ability to embed 3D models into websites and VLEs, such as CANVAS, enhance workflow efficiency and user engagement. We believe that our method adds to previously similar published work (Titmus et al., 2023), as it offers a comprehensive protocol from cadaver embalming to digitalisation, including image editing allowing natural colours to be brought back to the specimen texture, in addition to the ability to annotate and edit. Our protocol also uses embalming instead of plastination compared to other authors (Titmus et al., 2023), which is much less expensive to setup.

The next step to maximise the use of 3D educational models is through embedding them in immersive environments. Once finalised, our models can be embedded in VR, AR, or MR (all together identified as XR) to enhance the immersive experience of the 3D structure and its fruition. Embedding 3D anatomical models in XR for teaching may represent a significant advancement in medical education. This approach offers an immersive and interactive learning environment where students can engage with complex anatomical structures in a virtual space, enhancing their understanding and retention of information (Barmaki et al., 2019). An example of embedding one of our models into the real environment through the Sketchfab integrated AR module is presented in Figure 3i. According to some authors, VR and AR technologies in medical training can improve spatial understanding and procedural skills more effectively than traditional methods (Zhu et al., 2015). In addition, VR technology has been shown to improve student engagement, with a positive correlation also identified between student engagement levels and learning gain within higher education (Kazu & Kuvvetli, 2023; Ma et al., 2022). By integrating these 3D models into XR, educators can create collaborative and interactive learning experiences, allowing students to visualise and manipulate anatomical parts in real‐time, similarly to what has been developed in other fields (Gavish et al., 2015). The XR experience promises to provide a scalable and accessible platform, overcoming the limitations of physical cadavers and expensive simulation labs (Farrukh, 2024). It is beyond the scope of this manuscript to discuss in detail the advantages and disadvantages or the challenges offered by the XR; however, it is important to stress that the creation of anatomically correct 3D models is the foundation block of any anatomy‐related XR implementation and that this workflow can serve for the purpose of realising professional digital assets in this field. Despite the promising integration of 3D anatomical models in VR and MR for educational purposes, current haptic technologies present several limitations that hinder a fully immersive and realistic experience in virtual anatomy, which compare with the real experience. One significant challenge is the limited tactile feedback that current haptic devices can provide, falling short of replicating the complex feel and resistance encountered in real tissues and organs. This has not evolved substantially in the last decades (Srinivasan & Basdogan, 1997), nor has it reached a desired affordability to allow large‐scale deployment in teaching.

### 3.1 Limitations

The limitations associated with embalming techniques, including the WhitWell method, share some common grounds across all embalming methodologies (Brenner, 2014; Kaliappan et al., 2023). Several factors influence the quality of the embalmed specimens. One of the primary considerations is the condition of the cadaver, and timing of embalming is critical, as postmortem changes can impair the ability to achieve full perfusion due to blood clots blocking vessels. Ideally, embalming should occur within 24 h of death. If embalming cannot be done immediately, freezing the specimen as soon as possible and thoroughly thawing it before the procedure can mitigate some issues.

The size of the specimen can also present challenges. Although size does not directly affect the embalming process, specimens over 20 kg can pose significant manual handling difficulties. Another factor is body condition; lean specimens with well‐defined muscles are ideal because a high proportion of body fat can hinder the embalming process. Fat tissue is less vascularised, making it difficult for embalming fluids to penetrate fully. Additionally, obese specimens require more effort during dissection, as excess fat must be removed.

Species‐specific considerations are also important. Small domestic carnivores, such as dogs and cats, tend to embalm well despite the challenges mentioned. However, large domestic herbivores present more significant difficulties due to their large gut capacity, which often contains herbage that continues to ferment after embalming. This paper focuses solely on canine and feline embalming protocols, and a comprehensive analysis of the differences with larger herbivores is beyond its scope.

Handling embalmed specimens requires careful attention to formalin exposure. Formalin levels must be closely monitored throughout the embalming process to comply with health and safety regulations. Personal protective equipment (PPE) is essential. When formalin levels exceed legal limits, a ventilated hood or downdraft table should be used. At levels below this, protective aprons, gloves, and footwear are generally sufficient. Manual handling of specimens can also be challenging, particularly for those over 20 kg, which are more difficult to manage and store.

When preparing embalmed specimens for dissection, it is important to open the body cavities and drain any excess fluid. The specimen should then be thoroughly rinsed with cold water and left in a well‐ventilated area or on a downdraft table until the formalin fumes dissipate to safe working levels. Only then can the specimen be safely dissected.

Dissection itself presents further challenges. Most specimens require some level of dissection to expose the necessary anatomical structures, which can be a time‐consuming process. For instance, dissecting a detailed canine forelimb may take up to 20 h. While embalmed tissues resemble fresh tissues, they tend to be firmer in texture, duller in colour, and devoid of blood in the arteries and veins. These limitations should be clearly communicated to students; however, for teaching purposes, these limitations can be addressed through digitisation workflows, allowing for more lifelike representations in subsequent uses.

Storage is another important consideration for maintaining embalmed specimens. In this study, the specimens have been stored for up to 4 years, as the protocol was introduced in 2020. Properly embalmed specimens, however, can be stored for more than 10 years if they are carefully managed. Storage involves wrapping specimens in thick transparent polyethylene bags with a small amount of wetting solution to prevent tissue dehydration, as detailed in the methodology section. These bags must be tightly sealed to prevent evaporation, and the specimens should be stored in a refrigerator at 4°C. Regular checks are necessary to monitor for any signs of dehydration or mould, ensuring the long‐term viability of the specimens.

Creating digital 3D organs for anatomy education offers numerous advantages, as outlined before, such as accessibility and repeatability (Gianotto et al., 2023), but there are limitations compared to studying real human organs. In contrast with the unlimited availability of digital 3D models, not restricted by location and time after prosection, examination of real organs is crucial for students to experience and understand the original textures and consistencies of animal organs during undergraduate studies.

## 4 CONCLUSION

Selecting appropriate and innovative approaches in anatomy education involves multiple factors, including cadaver supply, appropriate cadaver preservation, curriculum design, sustainable storage infrastructure, digital capabilities, resource management, and pedagogical considerations. These elements collectively shape institutional strategies and cannot be considered in isolation.

During and after the COVID‐19 pandemic, The University of Liverpool, School of Veterinary Science has strategically adopted innovative approaches, focusing on advanced embalming techniques and the digitisation of anatomical specimens to ensure the sustainable continuity of anatomy education. We have successfully implemented the WhitWell‐Liverpool embalming method, which has received positive verbal feedback from students regarding the quality, appearance, and improved odour of the cadavers during teaching sessions. This embalming method has enabled us to conduct both prosection‐based teaching and dissection classes while also facilitating the creation of life‐like anatomical digital 3D models, which are often difficult to produce from fresh specimens. Although our current embalming practice offers several advantages over historic methods, this area remains open for further development. Future studies could refine the characteristics of anatomical specimens and enhance the learning experience for students.

The development of our technological infrastructure, including the newly established Digital Morphology Laboratory (DiMoLab), has enabled us to create and transform 3D anatomical models that are interactive and life‐like, supporting our students' educational needs. By utilising various software applications, we have developed a workflow which has allowed the creation of a digital library that can be accessed in any setting. Our future work will focus on expanding this library and collecting meaningful data on how this resource helps students achieve their learning outcomes and further develop and test their integration in immersive environments. The digitisation of anatomical and pathological specimens is an exciting and emerging field with the potential to transform the learning and teaching experience in education. It opens new avenues for research and has the capability to significantly enhance educational practices across health professions.

## AUTHOR CONTRIBUTIONS

Embalming (ZD), prosection of embalmed specimens (FP, ZD), acquisition, processing of images, and creation of 3D models (ZD, JA, LR, SC), photogrammetry workflow design (LR, ER), manuscript writing and editing (all authors), 3D model editing (LR), and project coordination (LR).

## CONFLICT OF INTEREST STATEMENT

Any information about the methodology in this paper may be requested by contacting the authors. The authors claim no conflicts of interest. Part of the contents of this paper were presented as an abstract and workshop format at the annual international Veterinary Education (VetEd) Symposium, Edinburgh, July 2023.

## Supporting information


**Table S1.** Summary of the WhitWell‐Liverpool Embalming Protocol and Chemicals.

**Video S1.** Video showing a 3D model of an anatomical specimen in motion.

## Data Availability

Data sharing not applicable to this article as no datasets were generated or analysed during the current study.

## References

[joa14192-bib-0001] Barmaki, R. , Yu, K. , Pearlman, R. , Shingles, R. , Bork, F. , Osgood, G.M. et al. (2019) Enhancement of anatomical education using augmented reality: an empirical study of body painting. Anatomical Sciences Education, 12(6), 599–609. Available from: 10.1002/ASE.1858 30648818

[joa14192-bib-0002] Brenner, E. (2014) Human body preservation—old and new techniques. Journal of Anatomy, 224(3), 316–344. Available from: 10.1111/JOA.12160 24438435 PMC3931544

[joa14192-bib-0003] Cheung, C.C. , Bridges, S.M. & Tipoe, G.L. (2021) Why is anatomy difficult to learn? The implications for undergraduate medical curricula. Anatomical Sciences Education, 14(6), 752–763. Available from: 10.1002/ASE.2071 33720515

[joa14192-bib-0004] Coleman, R. & Kogan, I. (1998) An improved low‐formaldehyde embalming fluid to preserve cadavers for anatomy teaching. Journal of Anatomy, 192(3), 443–446. Available from: 10.1046/J.1469-7580.1998.19230443.X 9688512 PMC1467790

[joa14192-bib-0005] Davis, H.C. & Fill, K. (2007) Embedding blended learning in a university's teaching culture: experiences and reflections. British Journal of Educational Technology, 38, 817–828.

[joa14192-bib-0006] de Lima, L.F.S. , de Barros, A.J.B.P. , Martini, A.D.C. , Stocco, M.B. , Kuczmarski, A.H. & de Souza, R.L. (2019) Photogrammetry and 3D prototyping: a low‐cost resource for training in veterinary orthopedics. Ciência Rural, 49(12), e20180929. Available from: 10.1590/0103-8478CR20180929

[joa14192-bib-0007] de Oliveira, A.S.B. , Leonel, L.C.P.C. , Lahood, E.R. , Hallak, H. , Link, M.J. , Maleszewski, J.J. et al. (2023) Foundations and guidelines for high‐quality three‐dimensional models using photogrammetry: a technical note on the future of neuroanatomy education. Anatomical Sciences Education, 16(5), 870–883. Available from: 10.1002/ASE.2274 36934316

[joa14192-bib-0008] Dixit, I. , Kennedy, S. , Piemontesi, J. , Kennedy, B. & Krebs, C. (2019) Which tool is best: 3D scanning or photogrammetry—it depends on the task. Advances in Experimental Medicine and Biology, 1120, 107–119. Available from: 10.1007/978-3-030-06070-1_9 30919298

[joa14192-bib-0009] Egels, Y. & Kasser, M. (2002) Image acquisition. Physical aspects. Instruments. In: Egels and Kasser (Eds.), Digital photogrammetry. London: Taylor & Francis, pp. 1–63.

[joa14192-bib-0010] Erolin, C. (2023) Preference for realism in 3D anatomical scans. Journal of Visual Communication in Medicine, 46(2), 85–96. Available from: 10.1080/17453054.2023.2226690 37395086

[joa14192-bib-0011] Evin, A. , Souter, T. , Hulme‐Beaman, A. , Ameen, C. , Allen, R. , Viacava, P. et al. (2016) The use of close‐range photogrammetry in zooarchaeology: creating accurate 3D models of wolf crania to study dog domestication. Journal of Archaeological Science: Reports, 9, 87–93. Available from: 10.1016/J.JASREP.2016.06.028

[joa14192-bib-0012] Farrukh, K. (2024) Metaverse in medical education: a paradigm shift. Pakistan Journal of Medical Sciences, 40(1), 255. Available from: 10.12669/pjms.40.1.8752 38196477 PMC10772407

[joa14192-bib-0013] Fruhstorfer, B.H. , Palmer, J. , Brydges, S. & Abrahams, P.H. (2011) The use of plastinated prosections for teaching anatomy—the view of medical students on the value of this learning resource. Clinical Anatomy, 24(2), 246–252. Available from: 10.1002/CA.21107 21322047

[joa14192-bib-0014] Gavish, N. , Gavish, N. , Gutiérrez, T. , Webel, S. , Rodríguez, J. , Peveri, M. et al. (2015) Evaluating virtual reality and augmented reality training for industrial maintenance and assembly tasks. Interactive Learning Environments, 23(6), 778–798.

[joa14192-bib-0015] Gianotto, I. , Coutts, A. , Pérez‐Pachón, L. & Gröning, F. (2023) Evaluating a photogrammetry‐based video for undergraduate anatomy education. Advances in Experimental Medicine and Biology, 1421, 63–78. Available from: 10.1007/978-3-031-30379-1_4/TABLES/2 37524984

[joa14192-bib-0016] Gilbert, J.K. (2004) Models and modelling: routes to more authentic science education. International Journal of Science and Mathematics Education, 2, 115–130.

[joa14192-bib-0017] Gurses, M.E. , Gonzalez‐Romo, N.I. , Xu, Y. , Mignucci‐Jiménez, G. , Hanalioglu, S. , Chang, J.E. et al. (2024) Interactive microsurgical anatomy education using photogrammetry 3D models and an augmented reality cube. Journal of Neurosurgery, 141(1), 17–26. Available from: 10.3171/2023.10.JNS23516 38277660

[joa14192-bib-0018] Hackmann, C.H. , dos Reis, D.A.L. & de Assis Neto, A.C. (2019) Digital revolution in veterinary anatomy: confection of anatomical models of canine stomach by scanning and three‐dimensional printing (3D). International Journal of Morphology, 37(2), 486–490. Available from: 10.4067/S0717-95022019000200486

[joa14192-bib-0019] Hayashi, S. , Naito, M. , Kawata, S. , Qu, N. , Hatayama, N. , Hirai, S. et al. (2016) History and future of human cadaver preservation for surgical training: from formalin to saturated salt solution method. Anatomical Science International, 91(1), 1–7. Available from: 10.1007/S12565-015-0299-5/TABLES/1 26670696

[joa14192-bib-0020] Hong, E.M. , Hakimi, A.A. , Ho, D. , Torkian, B.A. & Wong, B.J.F. (2021) Evaluating open source software for 3D imaging and morphing in cosmetic and reconstructive surgery. The Laryngoscope, 131(2), 299–303. Available from: 10.1002/LARY.28857 32710641

[joa14192-bib-0201] Kaliappan, A. , Motwani, R., Gupta, T., & Chandrupatla, M. (2023) Innovative Cadaver Preservation Techniques: a Systematic Review. Maedica (Bucur). 18(1), 127–135. Available from: 10.1007/S12565-015-0299-5/TABLES/1 PMC10231151 37266469

[joa14192-bib-0021] Kazu, İ.Y. & Kuvvetli, M. (2023) The impact of virtual reality technology on student engagement and learning outcomes in higher education. Available from: http://as‐proceeding.com/

[joa14192-bib-0022] Koh, M.Y. , Tan, G.J.S. & Mogali, S.R. (2023) Spatial ability and 3D model colour‐coding affect anatomy performance: a cross‐sectional and randomized trial. Scientific Reports, 13(1), 7879. Available from: 10.1038/S41598-023-35046-2 37188811 PMC10185657

[joa14192-bib-0023] Krause, K.J. , Mullins, D.D. , Kist, M.N. & Goldman, E.M. (2023) Developing 3D models using photogrammetry for virtual reality training in anatomy. Anatomical Sciences Education, 16(6), 1033–1040. Available from: 10.1002/ase.2301 37248365

[joa14192-bib-0024] Kumar, N. , Pandey, S. & Rahman, E. (2021) A novel three‐dimensional interactive virtual face to facilitate facial anatomy teaching using Microsoft HoloLens. Aesthetic Plastic Surgery, 45(3), 1005–1011. Available from: 10.1007/s00266-020-02110-5 33469701

[joa14192-bib-0025] Langfield, T. , Colthorpe, K. & Ainscough, L. (2018) Online instructional anatomy videos: student usage, self‐efficacy, and performance in upper limb regional anatomy assessment. Anatomical Sciences Education, 11(5), 461–470. Available from: 10.1002/ASE.1756 29205947

[joa14192-bib-0026] Laurillard, D. (2002) Rethinking university teaching: a framework for the effective use of educational technology, 2nd edition. London: Routledge/Falmer.

[joa14192-bib-0027] Ling, Y. , Li, C. , Feng, K. , Duncan, R. , Eisma, R. , Huang, Z. et al. (2016) Effects of fixation and preservation on tissue elastic properties measured by quantitative optical coherence elastography (OCE). Journal of Biomechanics, 49(7), 1009–1015. Available from: 10.1016/J.JBIOMECH.2016.02.013 26903410

[joa14192-bib-0028] Little, W.B. , Artemiou, E. , Conan, A. & Sparks, C. (2018) Computer assisted learning: assessment of the veterinary virtual anatomy education software IVALA™. Veterinary Sciences, 5(2), 58. Available from: 10.3390/VETSCI5020058 29921803 PMC6024603

[joa14192-bib-0029] Lombardero, M. , Yllera, M.M. , Costa‐e‐Silva, A. , Oliveira, M.J. & Ferreira, P.G. (2017) Saturated salt solution: a further step to a formaldehyde‐free embalming method for veterinary gross anatomy. Journal of Anatomy, 231(2), 309–317. Available from: 10.1111/JOA.12634 28542788 PMC5522894

[joa14192-bib-0030] Lone, M. , McKenna, J.P. , Balta, J.Y. , O'Mahony, S.M. , Cryan, J.F. , Downer, E.J. et al. (2017) Assessment of Thiel‐embalmed cadavers as a teaching tool for oral anatomy and local anesthesia. Journal of Dental Education, 81(4), 420–426. Available from: 10.21815/JDE.016.012 28365606

[joa14192-bib-0031] Ma, J. , Sun, C. & Wang, Y. (2022) The mediating role of learning engagement on learning gains of international students in Chinese higher education institutions—based on a multi‐cohort analysis. Sustainability, 14(21), 14052. Available from: 10.3390/SU142114052

[joa14192-bib-0032] Maresky, H.S. , Oikonomou, A. , Ali, I. , Ditkofsky, N. , Pakkal, M. & Ballyk, B. (2019) Virtual reality and cardiac anatomy: exploring immersive three‐dimensional cardiac imaging, a pilot study in undergraduate medical anatomy education. Clinical Anatomy, 32(2), 238–243. Available from: 10.1002/CA.23292 30295333

[joa14192-bib-0033] Marks, S.C.J. (2001) Recovering the significance of 3‐dimensional data in medical education and clinical practice. Clinical Anatomy, 14, 90–91. Available from: 10.1002/1098-2353 11135403

[joa14192-bib-0034] McLachlan, J.C. & Patten, D. (2006) Anatomy teaching: ghosts of the past, present and future. Medical Education, 40(3), 243–253. Available from: 10.1111/J.1365-2929.2006.02401.X 16483327

[joa14192-bib-0035] Nicolae, C. , Nocerino, E. , Menna, F. & Remondino, F. (2014) Photogrammetry applied to problematic artefacts. International Archives of the Photogrammetry, Remote Sensing and Spatial Information Sciences—ISPRS Archives, 40(5), 451–456. Available from: 10.5194/isprsarchives-XL-5-451-2014

[joa14192-bib-0036] Rajaram, A. , Fiset, P.O. & Fraser, R. (2023) Photogrammetry of “wet” pathology museum specimens: a pilot project. Collections: A Journal for Museum and Archives Professionals, 19, 536–552. Available from: 10.1177/15501906231189209

[joa14192-bib-0037] Remondino, F. , Barazzetti, L. , Nex, F. , Scaioni, M. & Sarazzi, D. (2012) UAV photogrammetry for mapping and 3D modeling: current status and future perspectives. The International Archives of the Photogrammetry, Remote Sensing and Spatial Information Sciences, XXXVIII‐1‐C22, 25–31. Available from: 10.5194/ISPRSARCHIVES-XXXVIII-1-C22-25-2011

[joa14192-bib-0038] Ricci, E. , Leeming, G. & Ressel, L. (2024) Photogrammetry: adding another dimension to virtual gross pathology teaching. Journal of Veterinary Medical Education, e20230159. Available from: 10.3138/JVME-2023-0159 39504188

[joa14192-bib-0039] Russell, J.E. , van Horne, S. , Ward, A.S. , Bettis, E.A. , Sipola, M. , Colombo, M. et al. (2016) Large lecture transformation: adopting evidence‐based practices to increase student engagement and performance in an introductory science course. Journal of Geoscience Education, 64(1), 37–51. Available from: 10.5408/15-084.1

[joa14192-bib-0040] Santana, L.I. , Buchaim, D.V. , Hamzé, A.L. , Bertoni, R.C. , de Henrique, S.B.C.R. , de Marchi, M.Â. et al. (2022) The history of anatomy, its importance and new trends in the teaching/learning process. Archives of Anatomy and Physiology, 7(1), 1–4. Available from: 10.17352/aap.000018

[joa14192-bib-0041] Srinivasan, M.A. & Basdogan, C. (1997) Haptics in virtual environments: taxonomy, research status, and challenges. Computers & Graphics, 21(4), 393–404.

[joa14192-bib-0042] Struck, R. , Cordoni, S. , Aliotta, S. , Pérez‐Pachón, L. & Gröning, F. (2019) Application of photogrammetry in biomedical science. In: Rea, P.M. (Ed.) Biomedical visualisation: volume 1. New York: Springer International Publishing, pp. 121–130. Available from: 10.1007/978-3-030-06070-1_10 30919299

[joa14192-bib-0044] Talevi, G. , Pannone, L. , Monaco, C. , Bori, E. , Cappello, I.A. , Candelari, M. et al. (2023) Evaluation of photogrammetry for medical application in cardiology. Frontiers in Bioengineering and Biotechnology, 11, 1044647. Available from: 10.3389/FBIOE.2023.1044647 36714012 PMC9879954

[joa14192-bib-0045] Tamayo‐Arango, L. & Garzón‐Alzate, A. (2018) Preservation of animal cadavers with a formaldehyde‐free solution for gross anatomy. Journal of Morphological Sciences, 35(2), 136–141. Available from: 10.1055/S-0038-1669434/ID/OR1211-28/BIB

[joa14192-bib-0046] Tayebinik, M. & Puteh, M. (2012) Blended learning or E‐learning? International Magazine on Advances in Computer Science and Telecommunications (IMACST), 3(1), 103–110.

[joa14192-bib-0047] Theoret, C.L. , Carmel, É.N. & Bernier, S. (2011) Why dissection videos should not replace cadaver prosections in the gross veterinary anatomy curriculum: results from a comparative study. Journal of Veterinary Medical Education, 34(2), 151–156. Available from: 10.3138/JVME.34.2.151 17446641

[joa14192-bib-0048] Thompson, B. , Green, E. , Scotcher, K. & Keenan, I.D. (2022) A novel cadaveric embalming technique for enhancing visualisation of human anatomy. Advances in Experimental Medicine and Biology, 1356, 299–317. Available from: 10.1007/978-3-030-87779-8_13 35146627

[joa14192-bib-0049] Titmus, M. , Whittaker, G. , Radunski, M. , Ellery, P. , de Oliveira, I.R. , Radley, H. et al. (2023) A workflow for the creation of photorealistic 3D cadaveric models using photogrammetry. Journal of Anatomy, 243(2), 319–333. Available from: 10.1111/JOA.13872 37432760 PMC10335382

[joa14192-bib-0050] Turchini, J. , Buckland, M.E. , Gill, A.J. & Battye, S. (2018) Three‐dimensional pathology specimen modeling using “structure‐from‐motion” photogrammetry: a powerful new tool for surgical pathology. Archives of Pathology & Laboratory Medicine, 142(11), 1415–1420. Available from: 10.5858/ARPA.2017-0145-OA 29846102

[joa14192-bib-0051] Turney, B.W. (2007) Anatomy in a modern medical curriculum. Annals of the Royal College of Surgeons of England, 89(2), 104–107. Available from: 10.1308/003588407X168244 17346399 PMC1964553

[joa14192-bib-0052] Varga‐Atkins, T. (2024) Multimodal learning: a practitioner guide. York: AdvanceHE.

[joa14192-bib-0053] Wesencraft, K.M. & Clancy, J.A. (2019) Using photogrammetry to create a realistic 3D anatomy learning aid with unity game engine. In: Paul M. Rea (Ed.), Advances in experimental medicine and biology, Vol. 1205. Berlin: Springer, pp. 93–104. Available from: 10.1007/978-3-030-31904-5_7 31894572

[joa14192-bib-0054] Young, C.P.L. & Perović, N. (2020) ABC LD—a new toolkit for rapid learning design. European Distance and E‐Learning Network, 1, 426–437.

[joa14192-bib-0055] Zhu, E. , Lilienthal, A. , Shluzas, L.A. , Masiello, I. & Zary, N. (2015) Design of mobile augmented reality in health care education: a theory‐driven framework. JMIR Medical Education, 1(2), e10. Available from: 10.2196/MEDEDU.4443 27731839 PMC5041345

[joa14192-bib-0056] Zilverschoon, M. , Kotte, E.M.G. , van Esch, B. , ten Cate, O. , Custers, E.J. & Bleys, R.L.A.W. (2019) Comparing the critical features of e‐applications for three‐dimensional anatomy education. Annals of Anatomy, 222, 28–39. Available from: 10.1016/j.aanat.2018.11.001 30465888

