# Supplementary material for: A complete workflow from embalmed specimens to life‐like 3D virtual models for veterinary anatomy teaching
Source: J Anat. 2024 Dec 20;246(5):857–68. doi: 10.1111/joa.14192 (PMC11996711; doi:10.1111/joa.14192)
Supplement: Supplementary file 1 — Table S1. Summary of the WhitWell‐Liverpool Embalming Protocol and Chemicals. [file JOA-246-857-s002.docx]

**Table 1 Summary of the WhitWell-Liverpool Embalming Protocol and Chemicals**

| **Task** | **Chemicals** | **Supplier** | **Instruments and Equipment** | **Protocol** |
| --- | --- | --- | --- | --- |
| **Joint manipulation** |  |  |  | Adequately mobilise all joints of the appendicular skeletal system via full range of motion to eliminate stiffness caused by types of rigor mortis. |
| **Disinfection** | ANIGENE (HLD4V, XTR106D)  dilution of 1:50  for 20 minutes | Byotrol Technology Ltd® | Brushes, washtubs | Trim the hair of dogs with thicker coats using clippers and shave the left ventro-lateral neck. Thoroughly scrub the entire body with disinfectant to remove dirt and dandruff. Manipulate the joints of the thoracic and pelvic limbs through their full range of motion to eliminate rigor mortis and improve embalming fluid penetration. |
| **Neck incision** |  |  | Scalpel,  Ligature cord,  Forceps,  Nasogastric (NG) tube (for vascular drainage), 12-18 Fr Aneurysm needle | - Make a ventral neck incision along the jugular groove to expose the external jugular vein (EJV) between the *sternocephalicus* and *cleidocephalicus* muscles. - Incise the EJV, insert a drainage tube, and secure it. Ligate the cranial end to prevent leakage. Use the EJV for draining blood and the carotid artery for administering embalming fluids. Both vessels are ligated cranially to prevent fluid leakage. - Through the same incision, expose the common carotid artery (CCA) via blunt dissection. Insert the cannula, secure it with ligatures, and tighten to prevent fluid leakage. |
| **Pre-fixation** | For up to 15 kg dog  Proflow (500 ml), [Datasheet](https://shop.dodgeco.com/dodge_website/static/src/doc/SDS/Proflow3.2021.pdf)  Rectifiant  (500ml)  [(Datasheet)](https://shop.dodgeco.com/dodge_website/static/src/doc/SDS/Rectifiant.pdf)  **For up to 30 kg dog, use; 1 L each* | Dodge, Billerica, MA 01821 | Automated embalming pump (LPP-EM524), Dodge, Billerica, MA 01821 | Mix pre-fixation reagents (anticoagulant) in the pump.  Secure canula with a ligature.  Pump 1 L of the pre-embalming fluid into the cannula and allow the blood to drain via the EJV.  Use pulse pressure 140–150 psi and flow rate 300-400 ml/min.  Extend and flex extremities to facilitate fluid movement. |
| **Embalming** | For upto 15 kg dog  Introfiant-(2 L),  Proflow- (1 L), [Datasheet](https://shop.dodgeco.com/dodge_website/static/src/doc/SDS/Proflow3.2021.pdf)  Rectifiant- (2 L), [(Datasheet)](https://shop.dodgeco.com/dodge_website/static/src/doc/SDS/Rectifiant.pdf)  Restorative- (1 L),  [(Datasheet)](https://shop.dodgeco.com/dodge_website/static/src/doc/SDS/Restorative3.2021.pdf)  Dis Spray- (1.5 L),  [(Datasheet)](https://shop.dodgeco.com/dodge_website/static/src/doc/SDS/DisSpray.pdf)  Halt GX- (0.5 L),  [(Datasheet)](https://shop.dodgeco.com/dodge_website/static/src/doc/SDScanada/English/EN_CAN_DOD-707_Halt_GX.pdf)  ---------------------  Total Mix- 8L  **For up to 30 kg dog, use; Introfiant- (3 L)* | Billerica, MA 01821 | Embalming pump (LPP-EM524), Dodge, Billerica, MA 01821 | Prepare the embalming fluid. Pump fluid at 120-140 psi and 300-400 ml/min flow rate. Watch for abdominal swelling, leg and tail rigidity, and tissue coloration in the ventral abdomen and ears. Manipulate limbs and extremities. Continue until tissues are fully coloured and filled. Add more fluid if needed.  Cannulate a local artery if extremities are poorly perfused. Decoagulated blood flushing through the jugular vein tube confirms successful embalming.  Abdomen swelling, and leg/tail rigidity demonstrates successful completion. |
| **Cannula withdrawal** |  |  | Ligature cord. Reverse triangular cut edge needle.  Artery forceps Toothed forceps Sutures | After the entire fluid has been pumped and the cadaver is fully embalmed, withdraw the cannula from the CCA, tighten the ligature, and tie it off.  Close the incision line with a baseball stitch using a needle, artery forceps, and toothed forceps. |
| **Disinfection and storage** |  |  | Body bags | Wash the body surfaces with disinfectant and spray the entire body with Dis Spray.  Transfer the cadaver into a large poly bag pre-sprayed with Dis Spray.  Move the cadaver to a refrigerated storage room (4°C) and store it for at least 8 weeks before dissection. |
| **Prosection and safety measures** | - **AFOS down draught Ventilated Dissection Table** - **Sundstrom SR 500 Powered Air Respirator** | - © 2024 AFOS. Wiltshire,Kingston upon, Hull, UK - Sundstrom, Sweden |  | - Specimens are dissected on ventilated downdraught tables to minimise exposure to formalin fumes and odours. - During cavity opening and fluid drainage, respiratory hoods (SR 500) are worn as part of PPE to handle high fume levels. - Once formalin levels drop below 2 ppm, specimens are moved to teaching rooms where fume levels are kept within safe limits. - Unlike conventional embalming fluids, Dodge chemicals have minimal lingering odour, as specimens are stored in sealed containers and the teaching rooms have high air circulation. |
